# Pediatric Foreign Body Ingestion: Analysis of Patient Characteristics and Surgical Treatment

**DOI:** 10.3390/children12101355

**Published:** 2025-10-09

**Authors:** Hee Jin Yeon, Sung Min Lee, Kyong Ihn, Jung-Tak Oh, Seok Joo Han, In Geol Ho

**Affiliations:** 1 Department of Surgery, Konyang University Hospital, Daejeon 35365, Republic of Korea; 200757@kyuh.ac.kr; 2 Division of Pediatric Surgery, Severance Children’s Hospital, Department of Surgery, Yonsei University of College of Medicine, Seoul 03722, Republic of Korea; panacea@yuhs.ac (S.M.L.); pedsik@yuhs.ac (K.I.); jtoh@yuhs.ac (J.-T.O.); sjhan@yuhs.ac (S.J.H.)

**Keywords:** pediatric foreign body ingestion, surgical intervention, magnets and water beads, gastrointestinal complications

## Abstract

**Highlights:**

**What are the main findings?**

- Surgical treatment was more frequently required in cases involving multiple FB ingestion, unwitnessed ingestion, and symptomatic presentation
- Magnets and water beads beyond the stomach were strongly associated with the need for surgical intervention

**What is the implication of the main finding?**

- The findings may assist pediatric surgeons in identifying high-risk patients and making timely decisions regarding surgical management
- Preventive measures, including caregiver education and public awareness, are crucial to reduce the incidence of hazardous FB ingestion in children

**Abstract:**

Background/Objectives: Foreign body (FB) ingestion is a common occurrence in children. We aimed to investigate the characteristics of patients hospitalized in the pediatric surgery department for FB ingestion and those requiring surgical treatment. Methods: We retrospectively analyzed pediatric patients admitted to the department of pediatric surgery at a single center for FB ingestion from June 2018 to September 2023. Overall, 35 patients were included. Results: The median age was 25.0 months (range: 7–204 months), with male predominance. Forty percent (14/35) of patients presented with symptoms at admission, including abdominal pain, vomiting, and fever; ingestion was unwitnessed in 42.9%. The median number of ingested FBs was 2.0 (range: 1–25), with magnets being the most common type (51.4%). Surgery was required in 51.4% of cases due to complications or impaction. These cases showed significantly higher symptomatic presentation rates (72.2% vs. 5.9%, *p* < 0.01), unwitnessed ingestion (66.7 vs. 17.6%, *p* = 0.006), and more ingested FBs (median 5.0 vs. 1.0, *p* = 0.001). Magnet ingestion was more frequent in these patients. Conclusions: FB ingestion predominantly affects young male children. Magnet ingestion frequently requires surgical intervention, underscoring the importance of preventive measures and safer environments. Surgical treatment should be considered in patients with symptomatic presentation, multiple ingested objects, or an uncertain ingestion history.

## 1. Introduction

Foreign body (FB) ingestion is a common accident in children. The incidence of FB ingestion and the need for surgical management due to complications have been increasing [1]. Endoscopic retrieval may be considered if the FB is located in the esophagus or stomach and the procedure is feasible. However, if an FB passes into the small intestine, the patient is normally admitted to the pediatric surgery department for observation. Some of these patients require surgical management, whether for direct removal of the FBs or for treating complications, while others pass the FB naturally in their stool without the need for surgery [2,3]. According to previous studies, FBs are generally expelled through the gastrointestinal tract or removed endoscopically, with minimal risk of complications [3,4]. In approximately 10% of cases, FBs progress beyond the stomach [5], with surgical treatment needed in fewer than 1% of cases [6,7]. However, there have been no studies analyzing patients admitted to the pediatric surgery department for FB ingestion. Therefore, we aimed to analyze the characteristics of patients with ingested FBs that have passed through the stomach and who have been admitted to the pediatric surgery department, and delineate the clinical features associated with cases that required surgical treatment.

## 2. Materials and Methods

This single-center retrospective study was conducted from June 2018 to September 2023. The study included patients admitted to the pediatric surgery department through the emergency room or outpatient department, or referred from other hospitals due to FB ingestion. Patients who underwent endoscopic removal were excluded, as the aim of this study was to investigate cases in which the FB had passed beyond the stomach and could not be managed endoscopically. The medical records of the patients were reviewed to determine sex, age, comorbidities, presence of witnesses of the FB ingestion, symptoms such as abdominal pain, fever, and vomiting; surgical intervention (including method and findings) and postoperative hospitalization duration; and number, type, and location of FBs. In patients who underwent surgery, the FB location was defined as the location where the FB was found during surgery. In patients who did not undergo surgery, the FB location was defined as the location identified through imaging evaluation upon admission. The Institutional Review Board and Ethics Committee of Severance Hospital approved this study (approval date: 27 November 2023; approval number: 4-2023-1305). The requirement for informed consent was waived due to the retrospective nature of the study and the minimal risk to participants. Statistical analyses were performed using SPSS Statistics for Windows, version 18.0 (SPSS Inc., Chicago, IL, USA). Continuous variables are expressed as median values with ranges due to the relatively small sample size and potential non-normal distribution, and were compared using Student’s *t*-test. Categorical variables are presented as frequencies and percentages, and were compared using the chi-square test, or Fisher’s exact test when the expected cell counts were small. Statistical significance was set at *p* < 0.05.

## 3. Results

A total of 912 patients presented with FB ingestion. Of these, 77 (8.44%) patients underwent endoscopic removal, while 752 (82.46%) patients were managed by observation without endoscopy. There were 19 patients in whom endoscopy was performed but no FB was identified. All of these patients were observed without further intervention. Specifically, 37 patients were admitted to the pediatric surgery department for surgical observation or operative management. Of there, two who underwent endoscopic removal during admission were excluded. Thus, 35 patients were included in this study (Table 1). The median age was 25.0 months (range: 7–204), with a higher frequency of male patients. Among the patients presenting with symptoms upon hospital admission, 40% (14/35) presented with abdominal pain, vomiting, or fever. The incidence of witnessed FB ingestion was 57.1% (20/35 patients). The median number of ingested FBs was 2.0 (range: 1–25). FBs were classified according to type as magnets, water beads (superabsorbent polymers), metals (including toy nails and hairpins), and other substances (including discharged batteries and slime toys), with magnets being the most common. The most common location for the FB was the small bowel. Surgical intervention was performed in 51.4% of patients, either for FB removal or the management of complications, while in the remaining 48.6% the FB was removed with stool passage, confirmed through observation.

Table 2 compares the surgery and observation groups. No significant differences were observed in age (median months, 24.5 vs. 26.0, *p* = 0.128) or sex ratio (1:0.8 vs. 1:0.88, *p* = 0.877) between the two groups. However, the surgery group exhibited a higher prevalence of symptomatic patients upon admission (72.2% vs. 5.9%, *p* < 0.01) and significantly fewer cases of witnessed FB ingestion (33.3% vs. 82.4%, *p* = 0.006). Additionally, the number of ingested FBs was significantly higher in the surgery group (median, 5 vs. 1; *p* = 0.001).

**Figure 1:**
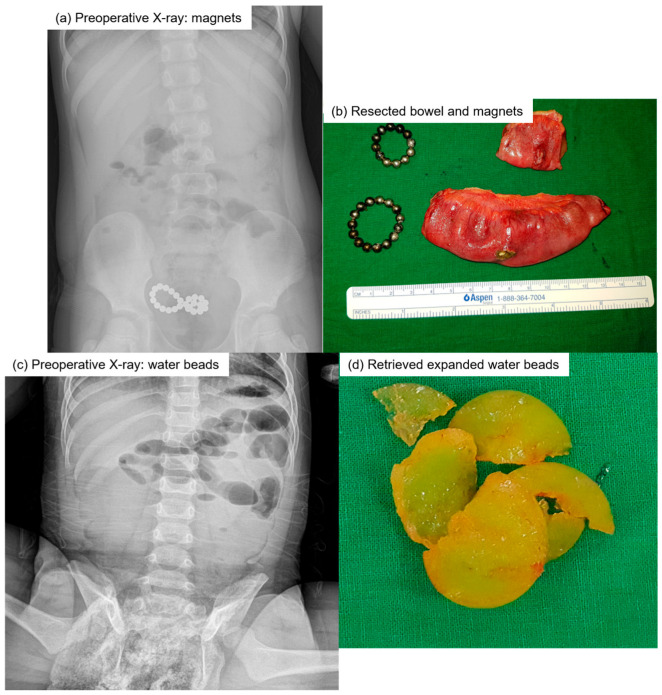
Representative cases of pediatric FB ingestion requiring surgical intervention. (**a**) Preoperative abdominal radiograph showing multiple magnetic beads clustered in the lower abdomen. (**b**) Resected small bowel segments and magnetic bead rings. The magnets induced pressure necrosis and inter-loop fistula formation. (**c**) Preoperative abdominal radiograph showing bowel dilatation consistent with small bowel obstruction. (**d**) Intraoperative photograph of retrieved superabsorbent polymer beads expanded within the intestinal lumen. FB, foreign body.

In the surgery group, the median time from admission to surgery was 0.5 days (range: 0–7), while the median time from surgery to discharge was 6.2 days (range: 3–11) (Table 3). Laparoscopic surgery was performed in 88.9% of cases (16/18), with conversion to laparotomy occurring in 11.1% of cases (2/18). Surgical findings revealed fistula formation, free perforation, and impaction alone in 44.4% (8/18), 16.7% (3/18), and 38.9% (7/18) of cases, respectively (Figure 1). Impaction refers to cases where FBs obstruct or become lodged in the bowel without causing complete obstruction. Segmental bowel resection, primary repair, FB removal after enterotomy, and manual dislodgement were performed in 33.3% (6/18), 22.2% (4/18), 38.9% (7/18), and 5.6% (1/18) of cases, respectively.

**Figure 2:**
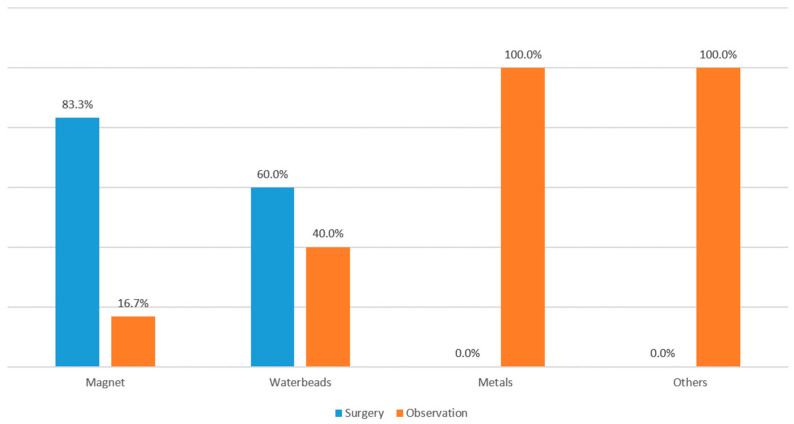
Proportion of surgical and observation cases by type of ingested FB. Magnets and water beads ingestions were more likely to require surgery, while all metal and other FB cases were managed conservatively. FB, foreign body.

Figure 2 illustrates the surgical intervention rates according to the type of FB ingested. Surgical treatment was performed in 83.3% and 60.6% of cases involving magnet and water bead ingestions, respectively. Most patients included in the study were infants, though there were also those aged 15–17 years. A 15-year-old patient ingested a FB while playing with friends, while a 17-year-old patient with a history of intellectual disability and bipolar disorder also presented with FB ingestion. Another patient with a psychiatric comorbidity (a 60 month-old child with autism) was included in the study.

## 4. Discussion

Accidental FB ingestion typically occurs during the oral exploration phase, which begins in infants over 6 months of age [6,8]. The highest incidence is observed between 4.5 and 5 years of age and tends to be more prevalent among male children [9,10]. However, our study, which examined patients admitted specifically to the pediatric surgery department, found a lower median age of 25 months and a male-to-female ratio of 1:0.53, indicating a younger cohort than those reported in the general literature on FB ingestion. Previous studies have reported that approximately 1% of pediatric patients presenting with FB ingestion required surgical intervention [10,11]. In our study, 1.97% of patients ultimately underwent surgery.

Previous studies have either focused on all patients presenting with FB ingestion or focused on a single type of FB, such as magnets or button batteries [2,6,12]. In contrast, this study is distinct in that it analyzed only pediatric patients in whom the FB had passed beyond the stomach, where endoscopic removal was not feasible, and surgical management needed to be considered. Notably, by comparing the characteristics of patients who ultimately required surgery, this study highlights specific predictive factors that may help identify those in need of surgical intervention.

This study identified FB ingestion itself and the presence of clinical symptoms as key factors associated with the need for surgical intervention. In clinical practice, early surgical intervention should be considered in cases of symptomatic presentation, ingestion of multiple FBs, or unwitnessed ingestion. In particular, when magnets or water beads have been ingested and endoscopic removal is not feasible, prompt surgical management is strongly recommended. Current NASPGHAN guidelines provide only limited recommendations, such as referring symptomatic patients with multiple magnet ingestion to the pediatric surgeon, and while water beads are mentioned, no clear management pathway has yet been established [2]. Our findings, therefore, provide additional, practical insights that may be of help to pediatric surgeons in their decision-making.

Previous research has indicated a link between unwitnessed ingestion and symptomatic presentation [13]. In agreement with this, we found that 73.3% of patients with unwitnessed ingestion exhibited symptoms upon admission to the hospital, whereas only 15.0% of those with witnessed ingestion were symptomatic (*p* < 0.01), demonstrating a statistically significant correlation (Table S1). Further multivariate analysis is warranted to determine additional predictors influencing the decision for surgery. Although the results did not reach statistical significance, surgical patients with unwitnessed ingestion exhibited a higher incidence of peritonitis (8 out of 12, 66.7%) than those with witnessed ingestion (1 out of 6, 16.7%). Notably, impaction was the most common surgical finding in witnessed cases (3 out of 6, 50%; Table S2). Given that peritonitis typically carries a worse prognosis than simple obstruction or impaction, these findings underscore the clinical importance of identifying FB ingestion early through direct observation. A prior study demonstrated that prolonged FB impaction increases the risk of complications, highlighting the potential benefit of early detection and timely intervention in reducing the likelihood of surgery for complication management [14].

Although coins are frequently reported as the most commonly ingested objects [6,8,9,10], magnets were the leading cause of surgical intervention in our study. Among the 15 surgical cases related to magnet ingestion, 11 involved peritonitis or the development of enteric fistulas. When multiple magnets are ingested at different times or locations, they may attract each other across intestinal loops, compressing the intervening walls and ultimately causing necrosis, perforation, or fistula formation [15]. In this study, cases of magnet ingestion that passed through the gastrointestinal tract without requiring surgery involved either a single or multiple magnets that remained clumped together as a single unit.

Water beads, composed of superabsorbent polymers, can expand up to 400 times their original volume upon water absorption. These items are frequently found in toys, gardening supplies, floral arrangements, and agricultural products. In contrast to most FBs, water beads continue to enlarge after ingestion, posing a delayed risk of small bowel obstruction even after passing the pylorus [16,17]. In our study, three of the five cases involving water beads required surgical treatment due to obstruction.

Previous studies have identified children with neurological or psychiatric conditions, such as developmental delay, autism, or behavioral disorders, as being at increased risk for FB ingestion [18,19,20]. In our study, two such cases were documented. One involved a 68-month-old boy with autism who ingested two magnets, which remained attached; he was managed conservatively and discharged without surgery. The other case was a 17 year-old girl with intellectual disability and bipolar disorder who ingested a metallic object and was similarly discharged after observation. Both patients were above the median age observed in the overall cohort. Previous research has also suggested that children with neurological or psychiatric conditions are not only at higher risk of FB ingestion but may also demonstrate distinctive patterns, such as repeated episodes of ingestion, a tendency to swallow unusually large or elongated objects, and a persistence of FB ingestion behavior into adolescence [20,21,22]. Although large-scale studies specifically addressing FB ingestion in pediatric patients with neurological or psychiatric conditions are lacking, this subgroup should be recognized as high-risk and warrants further investigation. Moreover, caregivers and educators who interact with these children would benefit from education and clear guidelines to help prevent such incidents and to provide targeted supervision

Laparoscopic surgery was the preferred approach for managing complicated FB ingestion in our study. In most cases, FBs were retrieved either via enterotomy or segmental resection of the small intestine. No postoperative complications were noted. These findings align with those of previous reports demonstrating that laparoscopy is a safe and effective alternative to open surgery for such cases [3,12].

This study had some limitations. First, it was a retrospective review conducted at a single institution, which may limit the generalizability of its findings. Second, the sample size was relatively small. Third, cases involving unwitnessed FB ingestion may be subject to recall bias. Finally, long-term postoperative outcomes were not evaluated. Future multicenter studies with larger patient populations and extended follow-up periods are needed to validate and expand upon these findings.

Collectively, this study provides important insights into the clinical characteristics and surgical indications of pediatric patients presenting with FB ingestion beyond the stomach. The findings underscore the importance of proactive preventive measures, including public awareness campaigns and the implementation of safer environmental regulations, to reduce the risk of hazardous FB ingestion. Future multicenter studies with long-term follow-up are warranted to enhance the generalizability of these results and to establish evidence-based guidelines that can assist pediatric surgeons in determining the need for surgical intervention. Additionally, further research into effective preventive strategies and the identification of risk factors for complications, particularly in patients with psychiatric conditions, may contribute to the development of more refined clinical guidelines for pediatric FB ingestion.

## 5. Conclusions

This study suggests that surgical treatment should be prioritized in cases involving the ingestion of magnets and water beads, multiple FBs, or unwitnessed ingestion accompanied by symptoms such as abdominal pain, fever, or vomiting.

## Figures and Tables

**Table 1:** Patient’s demographics (n = 35).

Variable		
Age (median, months)		25.0 (range 7–204)
Sex (M:F)		1:0.53
Symptomatic		14 (40.0%)
Unwitnessed		15 (42.9%)
Number of FB (median)		2.0 (range 1–25)
Type of FB	Magnet	18 (51.4%)
	Water beads ^1^	5 (14.3%)
	Metal	10 (28.6%)
	Others ^2^	2 (5.7%)
Location of FB	Small bowel	26 (74.3%)
	Colon	8 (22.9%)
	Stomach	1 (2.9%)
Operation		18 (51.4%)

^1^ water bead is a superabsorbent polymer. ^2^ including discharged batteries and slime toys.

**Table 2:** Comparison of the surgery group and the observation group.

	Surgery (n = 18)	Observation (n = 17)	*p* Value
Age (median, months)	24.5 (7–64)	26.0 (10–204)	0.128
Sex (M:F)	1:0.80	1:0.88	0.877
Symptomatic	13 (72.2%)	1 (5.9%)	<0.01
Unwitnessed	12 (66.7%)	3 (17.6%)	0.006
Number of FBs (median)	5 (1–25)	1 (1–4)	0.001

**Table 3:** Characteristics of the surgery group (n = 18).

Characteristics		
Median days from admission to surgery		0.5 (0–7)
Median days from surgery to discharge		6.2 (3–11)
Surgical approach	Laparoscopy	16 (88.9%)
	Conversion from laparoscopy to laparotomy	2 (11.1%)
Surgical findings	Fistula	8 (44.4%)
	Free perforation	3 (16.7%)
	Impaction alone	7 (38.9%)
Surgical procedure	Segmental resection	6 (33.3%)
	Primary repair	4 (22.2%)
	FB removal after enterotomy	7 (38.9%)
	Manual dislodgement	1 (5.6%)

## Data Availability

The data supporting the findings of this study are available from the corresponding author upon reasonable request.

## Supplementary Materials

The following supporting information can be downloaded at https://www.mdpi.com/article/10.3390/children12101355/s1: Table S1: Association between witnessed foreign body ingestion and presence of symptoms; Table S2: Association between witnessed foreign body ingestion and surgical findings.

## Glossary

Foreign Body (FB) Ingestion	The act of swallowing an object that is not food can cause complications if it becomes lodged in the gastrointestinal tract.
Endoscopic Removal	A non-surgical procedure where a flexible tube with a camera (endoscope) is used to visualize and remove foreign objects from the esophagus or stomach.
Surgical Intervention	The use of surgical procedures to treat or remove foreign bodies that cannot be extracted through endoscopy or when complications arise.
Small Intestine	A long, coiled tube in the digestive system where most of the digestion and nutrient absorption occurs, beyond the stomach.
Unwitnessed Ingestion	When a caregiver or medical personnel does not observe the ingestion of a foreign body, it makes it harder to determine the exact time of ingestion.
Superabsorbent Polymers (Water Beads)	Small, gel-like beads that expand dramatically when they absorb water are often used in toys and crafts. They can pose risks when ingested, as they may expand in the stomach or intestines.
Impaction	A condition where a foreign body becomes stuck or lodged in the gastrointestinal tract, causing obstruction or partial obstruction.
Laparoscopic Surgery	A minimally invasive surgical technique where small incisions are made, and a camera and instruments are used to perform the surgery, often preferred for its quicker recovery times than traditional open surgery.
Laparotomy	A more invasive surgical procedure that involves making a larger incision in the abdominal wall to access the organs is frequently used when laparoscopic surgery is not feasible.
Peritonitis	An infection or inflammation of the peritoneum, the lining of the abdominal cavity, often results from a perforation (hole) caused by ingested foreign bodies.
Fistula Formation	The development of an abnormal connection or passage between two organs or between an organ and the outside of the body, often a complication of foreign body ingestion.
Enterotomy	A surgical procedure where an incision is made in the intestine to remove a foreign body or to relieve an obstruction.
Segmental Bowel Resection	A surgical procedure where a portion of the intestine is removed, typically used when there is significant damage or obstruction.
Superabsorbent Polymers	Materials that can absorb and retain large amounts of liquid are used in products, such as diapers and water beads, which pose risks if ingested due to their expanding properties in the digestive system.
Developmental Delay	A condition where a child does not reach developmental milestones (e.g., speaking and walking) at the expected age, which may increase the risk of foreign body ingestion.
Behavioral Problems	Conditions such as autism, attention-deficit hyperactivity disorder, or other behavioral disorders may increase the likelihood of foreign body ingestion in children.
FB	Foreign body
