# Supplementary material for: Pediatric Foreign Body Ingestion: Analysis of Patient Characteristics and Surgical Treatment
Source: Children (Basel). 2025 Oct 9;12(10):1355. doi: 10.3390/children12101355 (PMC12563314; doi:10.3390/children12101355)
Supplement: Supplementary file 1 [file children-12-01355-s001.zip › children-3832554-supplementary.pdf]

Supplementary Material

Supplement 1. Association between witnessed foreign body ingestion and presence of symptoms		
	Symptomatic	Asymptomatic
Unwitnessed (n=15)	11 (73.3%)	4 (26.7%)
Witnessed (n=20)	3 (15.0%)	17 (85.0%)

Supplement 2. Association between witnessed foreign body ingestion and surgical findings			
	Peritonitis	Obstruction	Impaction
Unwitnessed (n=12)	8 (66.7%)	1 (8.3%)	3 (25.0%)
Witnessed (n=6)	1 (16.7%)	2 (33.3%)	3 (50.0%)
